# Identification of PP2C gene family and its role in stress and adversity based on T2T flax (*Linum usitatissimum* L.) genome

**DOI:** 10.1186/s12870-025-07284-1

**Published:** 2025-10-21

**Authors:** Jianyu Lu, Hang Wang, Jinxi Li, Hanlu Wu, Yifei Wang, RuiDong Sun, Michael K. Deyholos, Xiaonan Wang, Jun Zhang, Yan Gu, Jian Zhang

**Affiliations:** 1 Faculty of Agronomy, Jilin Agricultural University, Changchun, 130118 China; 2 Changchun International Biotechnology Laboratory for Fiber Plants, Changchun, 130118 China; 3 Department of Biology, University of British Columbia, Okanagan, Kelowna, BC V5K1K5 Canada; 4 Insitutute of Fiber Plant, Heilongjiang Academy of Science, Daqing, China

**Keywords:** Flax, *PP2C*, Bioinformatics, Expression analysis, Abiotic stress

## Abstract

The PP2C gene family plays crucial roles in plant growth, development, and responses to biotic/abiotic stresses. Flax (*Linum usitatissimum* L.), as an important oilseed and fiber crop, lacks comprehensive characterization of its *PP2C* genes. In this study, we conducted a genome-wide bioinformatics analysis of the *PP2C* gene family in flax using the Telomere to Telomere (T2T) genome assembly, identifying 117 *LuPP2C* genes. Phylogenetic analysis classified these *LuPP2C* proteins into 11 distinct subclades. Gene structure and motif analyses revealed conserved exon-intron architectures and motif compositions among members within the same phylogenetic branches. Cis-regulatory element analysis of *LuPP2C* promoters identified abundant stress-responsive elements, including those associated with plant hormones (MeJA and ABA) and abiotic stresses (anaerobic induction, drought responsiveness, and low-temperature adaptation). Genomic duplication events revealed 104 segmental duplication pairs, suggesting expansion through large-scale duplication. miRNA target prediction identified lus-miR395 as the predominant miRNA targeting *LuPP2C* family members. Expression profiling demonstrated preferential expression of most *LuPP2C* members in leaf tissues. Quantitative real-time PCR (qRT-PCR) analysis further revealed that subfamily A genes, particularly *LuPP2C26* and *LuPP2C99*, were significantly upregulated under cold, drought, and salt stress conditions. Functional validation through heterologous expression confirmed that overexpression of *LuPP2C26* and *LuPP2C99* enhances salt tolerance in yeast transformants. These findings systematically characterize the flax *PP2C* family and provide insights into its potential roles in stress adaptation mechanisms.

**Supplementary Information:**

The online version contains supplementary material available at 10.1186/s12870-025-07284-1.

## Introduction

Under abiotic stresses such as drought, salinity, and heat shock, plants undergo physiological and metabolic perturbations that elicit a cascade of ionic homeostasis disturbances. These stress conditions induce dynamic imbalances in plasma membrane potential, disrupt the regulatory network of endogenous hormone homeostasis, and impair multi-tiered cascade signaling systems spanning from plasma membrane-based stress perception to intracellular signal transduction [1]. Plants perceive environmental stimuli and orchestrate adaptive responses to abiotic stresses through sophisticated signal transduction cascades. Post-translational phosphoregulation – particularly reversible protein phosphorylation mediated by counterbalanced kinase-phosphatase systems–constitutes a fundamental regulatory mechanism in plant cellular signaling networks [2]. Protein phosphatases (PPs) remove phosphoryl modifications from phosphorylated proteins, thereby regulating stress responses, transducing abscisic acid (ABA) signals, and enhancing plant immunity [3, 4]. The PPP phosphatase superfamily comprises various catalytic subunit variants, including canonical members such as PP1, PP2A, PP2B, and PP4-PP7, while PP2C-type phosphatases exhibit the greatest family diversity in the plant kingdom. Notably, despite functional associations with PPP family members, *PP2C* phosphatases are classified into the distinct PPM superfamily, as their catalytic domains lack significant primary structural conserva [5, 6]. *PP2C* is a multifunctional protein phosphatase characterized by a distinctive architecture: a conserved catalytic domain at the C-terminus and variable-length extension regions at the N-terminus. The structural heterogeneity of these extension regions underlies *PP2C’s* functional versatility in diverse biological processes [6].

Members of the PP2C gene family mediate diverse physiological processes in plants, spanning abscisic acid (ABA) signaling transduction, developmental programming, wound-responsive jasmonate signaling, and systemic acquired resistance (SAR) activation [7]. PP2C-A subfamily phosphatases are identified as core regulatory components of the ABA signal transduction pathway in phylogenetic taxonomy. The promoter regulatory modules of nine homologous PP2C-A subfamily genes in *Arabidopsis* have evolutionarily conserved ABRE (ABA-responsive element) binding domains. Functional genomics studies reveal that canonical members such as *ABI1*, *PP2CA*, and *HAB1/2* exert negative regulatory functions in the ABA signaling pathway within molecular regulatory networks through phosphorylation cascades [8]. Multiple members of the *PP2C* gene family subfamily B negatively regulate the mitogen-activated protein kinase (*MAPK*) pathway, while protein phosphorylation and dephosphorylation are involved in all physiological processes in plants [9]. In *Arabidopsis*, *AP2C3* specifically regulates the expression of genes including *MPK3*, *MPK4*, and *MPK6*, while the *MAPK* signaling pathway induces epidermal pavement cell differentiation, thereby suppressing stomatal cell development [10]. Overexpression of *NtMPK4* in tobacco hairy roots and transient expression of *NtMPK4* in tobacco promote nicotine synthesis, while NtPP2C2b suppresses nicotine accumulation [11]. Members of the C subfamily, *POLTERGEIST* (*POL*) and *POL1–5* (*PLL1–5*), act as negative regulators of the *CLAVATA* (*CLV1*) signaling pathway, and are involved in the development of vegetative tissues and floral meristems [12]. Members of the D subfamily regulate plant growth and development by physically interacting with *SMALL AUXIN UP RNA* (*SAUR*) proteins and plasma membrane (PM) H⁺-ATPases to inhibit cell expansion [13]. The PP2C gene modulates abiotic stress signaling in plants, conferring multistress tolerance to drought, thermic extremes, salinity, and chilling [14, 15]. *Arabidopsis* E subfamily *PP2C6-6* and G subfamily *PP2CG1* can positively regulate *Arabidopsis* salt stress response [14, 16]. Additionally, the phosphorylation-dephosphorylation regulatory feedback loop mediated by *PP2CG1* phosphatase and *OOST1* protein kinase participates in regulating the plant’s cold stress response [17]. While the PP2C gene family has been widely investigated in diverse plant species including rice [18], maize [19], *Apocynum venetum* [20], and cucumber [21], with its functions in plant growth, development, and stress tolerance gradually elucidated, a comprehensive characterization in flax remains lacking.

Flax, a paleo-domesticated crop cultivated globally with temperate latitude predominance [22], exhibits agronomic classification based on utilization: linseed (oil), bast fiber (textile), and dual-purpose cultivars [23, 24]. Flax seeds contain bioactive compounds including lignans and soluble fiber, with α-linolenic acid (ALA) serving as a principal plant-derived ω−3 fatty acid precursor essential for human biosynthesis of eicosapentaenoic acid (EPA) and docosahexaenoic acid (DHA) [25]. This pioneering genome-wide investigation delineates the PP2C phosphatase family in flax, integrating phylogenomic profiling with structural characterization (domain architecture, sequence motifs, cis-regulatory elements), interactome mapping, and stress-responsive expression dynamics under cold, salinity, and drought regimes. This provides a foundation for further research on the biological functions of *PP2C* genes in flax. This work represents the first comprehensive investigation of *PP2C* gene family organization and stress-responsive regulation in flax.

## Materials and methods

### Plant material

The experimental system employed flax cv. ‘Gaosi’ subjected to standard surface-sterilization protocols (75% ethanol, 10 min) followed by triple-rinsing with autoclaved ddH₂O. Sterilized propagules were aseptically transferred to vermiculite-based growth substrate under controlled environmental conditions (16/8 h photoperiod, 25 ± 1 °C, 60% RH). Plants were cultivated in a growth chamber under 26 °C/18°C (day/night) temperatures with a 16-h light/8-h dark photoperiod. When flax seedlings reached 6–7 cm in height, stress treatments were applied: For drought and salt stress groups, plants were carefully removed from soil, thoroughly rinsed with distilled water, and transferred to conical flasks containing 10% polyethylene glycol (PEG-6000) and 100 mM NaCl solution, respectively. The control group was maintained in distilled water, while the low-temperature treatment group was placed in a 4 °C growth chamber. Leaf tissues from all treatment groups were simultaneously collected at 0, 3, 6, 12, and 24 h post-treatment, with three biological replicates per time point to minimize circadian rhythm effects. All biological specimens are frozen in liquid nitrogen within 30 s after collection and stored at −80 °C.

### Identification of PP2C gene in flax

The genomic sequence and annotation files were obtained from the unpublished telomere-to-telomere (T2T) genome of the flax cultivar ‘Gaosi’ generated by our research group. We retrieved 76 *Arabidopsis PP2C* protein sequences from the TAIR database (https://www.arabidopsis.org/) [26]. Candidate flax *PP2C* genes were identified by performing a Blastp alignment (E-value cutoff: 1e − 5) against the flax whole-genome protein sequences. The hidden Markov model (HMM) profile corresponding to the PP2C phosphatase catalytic domain (Pfam accession PF00481) was retrieved from the Pfam protein family database (http://pfam.xfam.org/) [27]. HMMER3.0 software with the hmmsearch program was further employed to predict flax *PP2C* genes. Candidate genes were filtered and validated using CDD (https://www.ncbi.nlm.nih.gov/cdd/) to confirm domain integrity. A total of 117 *LuPP2C* genes were ultimately identified, named with the prefix “Lu” followed by sequential numbering based on their chromosomal locations. Computational characterization of their physicochemical parameters—including coding sequence (CDS) length, amino acid residue count, molecular mass (Da), theoretical isoelectric point (pI), and grand average of hydropathicity (GRAVY) was performed using the ExPASy ProtParam platform (https://web.expasy.org/protparam/). The BUSCA net station was used to carry out the sub-cellular location pre-test (http://www.busca.cn).

### Phylogenesis, chromosome localization, conserved domains, and conserved motifs of LuPP2C gene

The PP2C amino acid sequences from *Arabidopsis*, flax, and related plants were aligned using ClustalW in MEGA v11 with default parameters. A phylogenetic tree was constructed via the maximum likelihood (ML) method in MEGA v11 under the JTT + G substitution model (parameters: 1,000 bootstrap replicates). The *LuPP2C* gene was located on the chromosome by flax genomic FASTA file and gff3 annotation file. Using MEME motif analysis of *LuPP2C* amino acid motifs (http://alternate.meme-suite.org/tools/meme), and visualization using TBtools (version 2.069) [28].

### Genome wide replication and collinearity analysis of LuPP2C gene

Genomic assemblies and annotation datasets for *Arabidopsis* along with maize, rice, and wheat were retrieved from Phytozome v13 (https://phytozome-next.jgi.doe.gov/) [29]. Collinearity relationships were predicted using the MCScanX toolkit. Whole-genome duplication (WGD)-derived *LuPP2C* genes were identified based on collinearity analysis. Segmental duplicated genes were detected via BLASTN (E-value cutoff: <1e − 5) by scanning 100-kb flanking regions (50 kb upstream and downstream) around coding sequences (CDS). Duplication events were confirmed if aligned sequences met the following criteria: alignment length ≥ 200 bp and sequence similarity > 85% [30].

### MiRNA prediction and cis-acting element analysis

Flax miRNA sequences were obtained from previous studies [31]. *LuPP2C* miRNA targets were computationally screened by aligning flax miRNAs with 5’/3’ untranslated regions (UTR) and coding sequences (CDS) using the psRNATarget platform (https://www.zhaolab.org/psRNATarget/analysis?function=3) [32]. The genomic sequences upstream (2000 bp) of all *LuPP2C* genes were extracted using TBtools software. Cis-regulatory elements were predicted via the PlantCARE online platform (https://bioinformatics.psb.ugent.be/webtools/plantcare/html/), and results were visualized using TBtools.

### Construction of protein–protein interaction network, validation of interacting proteins, and ontology enrichment (GO) enrichment analysis

To predict the protein-protein interaction network of the *LuPP2C* gene family, orthologous *Arabidopsis PP2C* genes corresponding to flax *PP2C* genes were used as reference. The functional PPI network was predicted using the STRING database (https://string-db.org/) with default parameters and visualized via Cytoscape software [33]. For Gene Ontology enrichment (GO) analysis of *LuPP2C* genes, the GO-base.ob file was downloaded through TBtools, and genome-wide flax protein sequences were annotated using the eggNOG-mapper online platform (http://eggnog-mapper.embl.de/). The resulting GO annotations were visualized using TBtools.

The CDS sequence of the *LuPP2C31* gene was ligated into the pGADT7 vector. The CDS sequence of the *LuPYL1* gene was cloned into the pGBKT7 vector. The recombinant plasmids LuPP2C31-AD and LuPYL1-BD were co-transformed into yeast strain Y2HGold competent cells. The transformed yeast cells were cultured on SD/-Trp/-Leu, SD/-Trp/-Leu/-Ade/-His, and SD/-Trp/-Leu/-Ade/-His + X-α-Gal media at 30 °C for 4 days, and colony formation was observed. The primers used for this assay are listed in Table S6.

### Expression pattern analysis of LuPP2C gene family

This study sequenced the transcriptomes of five flax species: (i) pistil, stamen, fruit, and shoot apical meristem tissues (PRJNA1002756), (ii) floral tissues at 30, 20, 10, and 5 days post-anthesis (PRJNA833557), (iii) embryo, anther, and seed tissues (PRJNA663265), (iv) root and leaf tissues under salt stress (PRJNA977728) [34], and (v) stem tissues under heat stress (PRJNA874329). Raw sequencing reads were quality-trimmed using fastp (v0.23.4), aligned to the telomere-to-telomere genome of flax ‘Gaosi’ with Rsubread (v2.14.2) [35], and processed for transcript quantification and expression analysis using tidyverse (v2.0.0) [36], edgeR (v3.42.4), and limma (v3.56.2) [37]. Three independent biological replicates were analyzed for each gene and tissue. Finally, a heatmap of log2(FPKM + 1) values was generated using TBtools.

### RNA extraction and fluorescence quantitative PCR analysis

Flax leaf tissue was crushed using a high-throughput tissue grinder (Scientz-48 L, Ningbo Scientz Biotechnology Co., Ltd, Ningbo, China). Total RNA isolation was performed using the BIOMGA RNA extraction kit (San Diego, USA). The SPARKscript Ⅱ RT Plus Kit (With gDNA Eraser) (Shandong Sparkjade Biotechnology Co., Ltd.) was utilised to create cDNA. Quantitative reverse transcription PCR (qRT-PCR) assays were conducted with the TB Green™ Premix Ex Taq™ II system (TaKaRa Bio, Kyoto, Japan), employing 20 µL reaction volumes containing gene-specific primers (Table S6) designed via Primer Premier 5.0 software. qRT-PCR was performed on independently prepared RNA samples for the selected genes. For each biological replicate, triplicate technical replicates were analyzed, with expression levels normalized against the endogenous reference gene GAPDH (glyceraldehyde-3-phosphate dehydrogenase). Relative expression was quantified using the 2^−ΔΔCt^ method [38].

### Heterologous expression of PP2C gene in the INVSc1 strain of brewing yeast

The *LuPP2C26* and *LuPP2C99* genes were directionally inserted into the multiple cloning site of the Saccharomyces cerevisiae expression vector pYES2 using restriction enzymes Hind III/Bam HI, resulting in recombinant plasmids pYES2-*LuPP2C26* and pYES2-*LuPP2C99*. These were transformed into INVSc1 yeast competent cells. INVSc1 yeast cells carrying the pYES2 empty vector served as the control. Yeast cells containing recombinant plasmids and controls were serially diluted to 10^−1^, 10^−2^, 10^−3^, and 10^−4^ concentrations. 10 µL aliquots of each dilution were spotted onto both SD-ura and SD-ura supplemented with 1 M NaCl solid media. The plates were incubated at 29 °C for 2–3 days, after which yeast growth was observed.

## Results

### Identification and phylogenetic analysis of members of the PP2C gene family in flax

A total of 117 *PP2C* genes were identified in the flax (Gaosi) genome based on the Hidden Markov Model (HMM) of the *PP2C* structural domain (PF00481). These genes were systematically named *LuPP2C1*–*LuPP2C117* according to their chromosomal locations (Table S1). Analysis of the predicted physicochemical properties revealed that *LuPP2C32* encodes the longest protein (1,091 amino acids), while *LuPP2C76* produces the shortest protein (186 amino acids). The molecular weights (MW) of *LuPP2C* proteins ranged widely from 20.00 kDa to 119.59 kDa. Notably, 78% of *LuPP2C* proteins exhibited an isoelectric point (pI) > 7, indicating a predominance of basic amino acids. The instability index varied between 29.43 and 62.43, with only 10.3% classified as stable proteins. The aliphatic index spanned 64.17–98.39, reflecting significant variability in thermostability among family members. Grand Average of Hydropathicity (GRAVY) values were universally negative (− 0.038 to − 0.568), confirming the hydrophilic nature of all *LuPP2C* proteins, though *LuPP2C76* (− 0.038) displayed near-neutral hydrophilicity. Bioinformatic subcellular localization predictions demonstrated exclusive nuclear compartmentalization of all *LuPP2C* gene products.

**Fig. 1:**
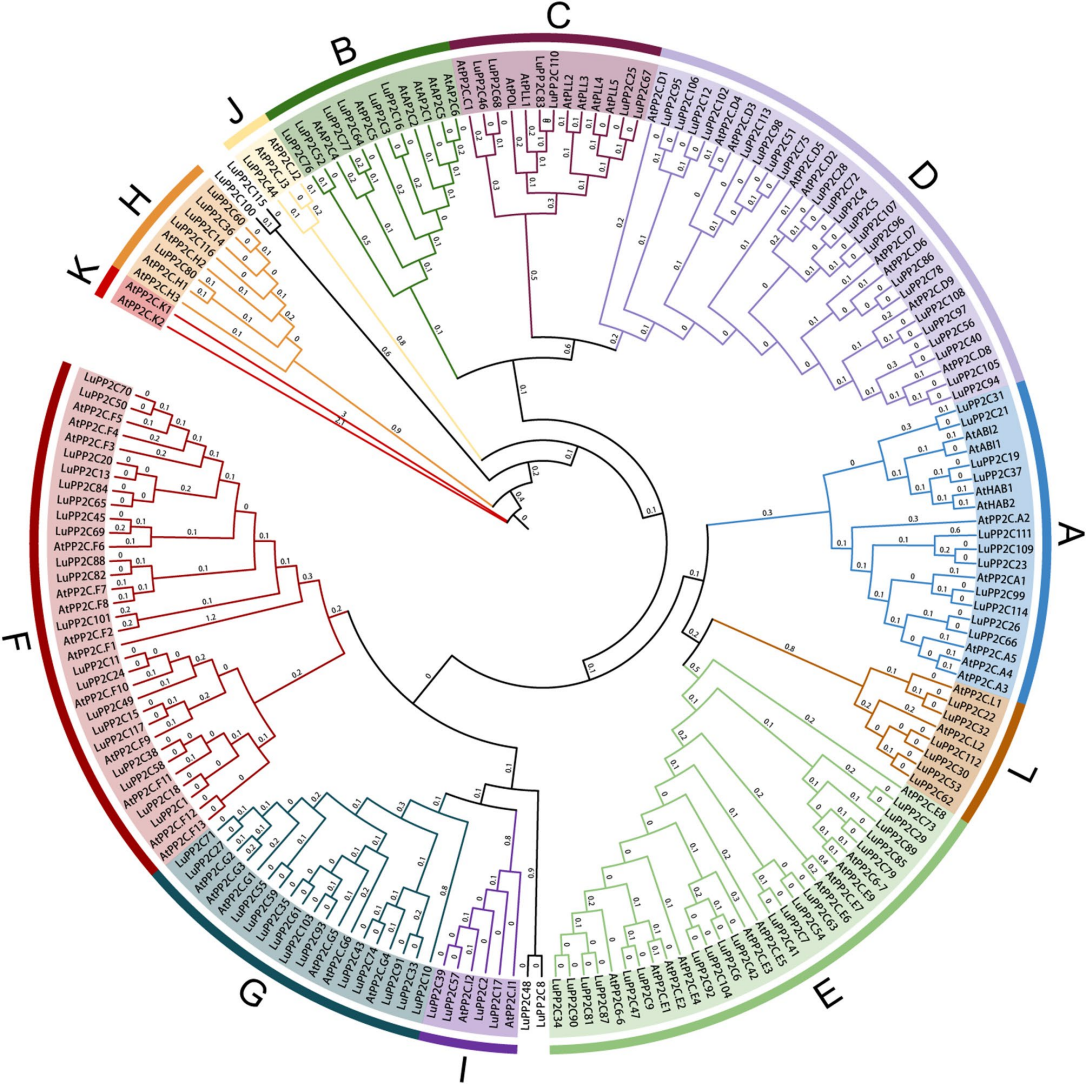
PP2C protein phylogenetic tree. The prefix At represents *Arabidopsis* genes, and the prefix Lu represents flax genes. All *PP2C* genes can be divided into 12 subfamilies, represented by different colors and letters

To elucidate the evolutionary dynamics of the *PP2C* gene family in flax, a maximum-likelihood phylogeny was reconstructed from 193 orthologs, comprising 76 *Arabidopsis* and 117 flax PP2C members, resolving clade-specific diversification patterns (Fig. 1) (Table S2). Based on the subfamily classification of *Arabidopsis PP2C* proteins, 113 *LuPP2C* genes (excluding *LuPP2C8*, *LuPP2C48*, *LuPP2C100*, and *LuPP2C115*) were divided into 12 subfamilies, named K, H, J, B, C, D, A, L, E, I, G, and F. No *LuPP2C* genes were found in the K subfamily, resulting in the distribution of 113 *LuPP2C* genes across 11 subfamilies. Among them, the D subfamily contained the largest number of *LuPP2C* genes (22), followed by the F subfamily with 20 *LuPP2C* genes, and the E subfamily with 19 members. The J subfamily had the fewest *LuPP2C* genes, containing only 1 gene. 

### Gene structure and conserved motif analysis of LuPP2C

**Fig. 2:**
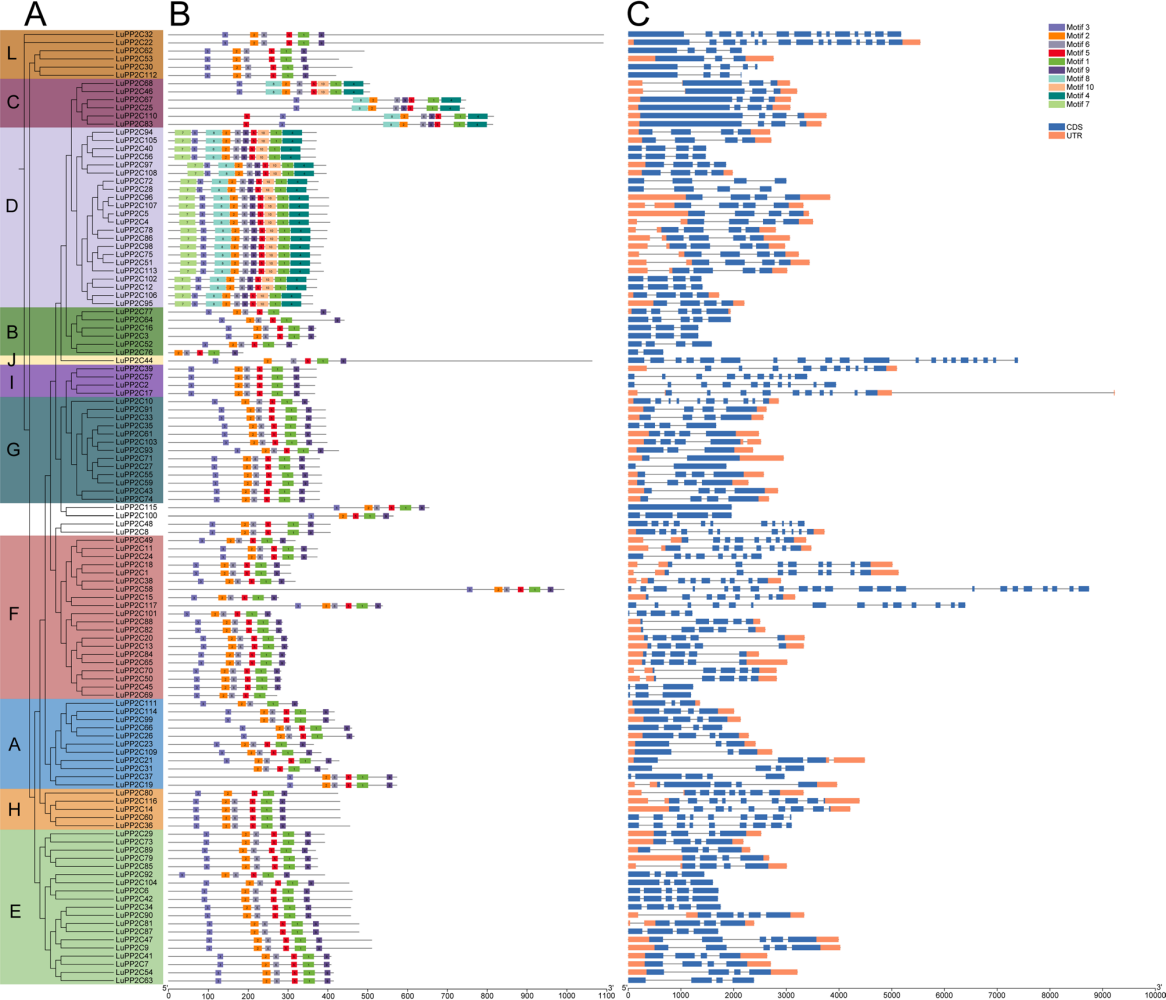
Genomic organization and evolutionary features of *LuPP2C* genes. **A** Maximum-likelihood phylogeny of *LuPP2C* orthologs. **B** Conserved motif architecture with color-coded functional domains. Genomic spans are denoted by gray connectors. **C** Exon-intron organization with 5′/3′ untranslated regions (UTR, orange), coding sequences (CDS, dark blue), and intervening intronic regions (black)

To analyze the structural characteristics of the flax *PP2C* gene family proteins, the conserved domains of the amino acid sequences of 117 *LuPP2C* genes were predicted. A phylogenetic tree was constructed from multiple sequence alignments (Fig. 2A), showing the same clustering as Fig. 1. The results predicted 10 conserved motifs, which were named motif1-motif10 (Fig. 2B). In addition, through the evaluation of motif 1–10 by pfam, it was found that only motif1-4 had a function related to the PPM-type phosphatase domain (Table S3). The amino acid length of the 10 motifs ranged from 15 to 41, and the sites ranged from 22 to 117 In all subfamilies, motif1, motif2, and motif5 are included, while motif4 only exists in the C and D subfamilies. All members except for *LuPP2C76* gene contain motif3, while only *LuPP2C80*, *LuPP2C100*, *LuPP2C46*, and *LuPP2C68* do not contain motif6 and motif9, while the rest of the members contain them. The motif type, number, and distribution of *LuPP2C* proteins under the same subfamily are relatively similar. For example, in the D subfamily, all members contain motif1-10, of which motif7 is a unique amino acid sequence type of the D subfamily. Although the exact function of motif7 remains unclear, its exclusive occurrence in D subfamily members may indicate a role in subfamily-specific regulatory mechanisms or interactions. The protein domains of different subfamilies of the *LuPP2C* gene exhibit minimal differences, further supporting the existence of shared biological functions across these subfamilies.

Analysis of the exon-intron structures of *LuPP2C* genes revealed that the number of exons ranged from 1 to 22, with introns varying between 0 and 21 (Fig. 2C). LuPP2C58 exhibited the highest number of exons (22) and introns (21), followed by *LuPP2C44* with 20 exons and 19 introns, while *LuPP2C115* contained only 1 exon. Although gene structures were generally conserved within subfamilies, significant variations in exon/intron numbers were observed in specific genes. For example, within the L subfamily, four genes contained 3–5 exons, whereas *LuPP2C22* and *LuPP2C32* harbored 15 exons. Notably, most genes within the same phylogenetic branch shared similar exon counts; all members of the D subfamily uniformly possessed 4 exons, suggesting functional conservation among these genes.

### Chromosome localization and collinearity analysis of LuPP2C gene

**Fig. 3:**
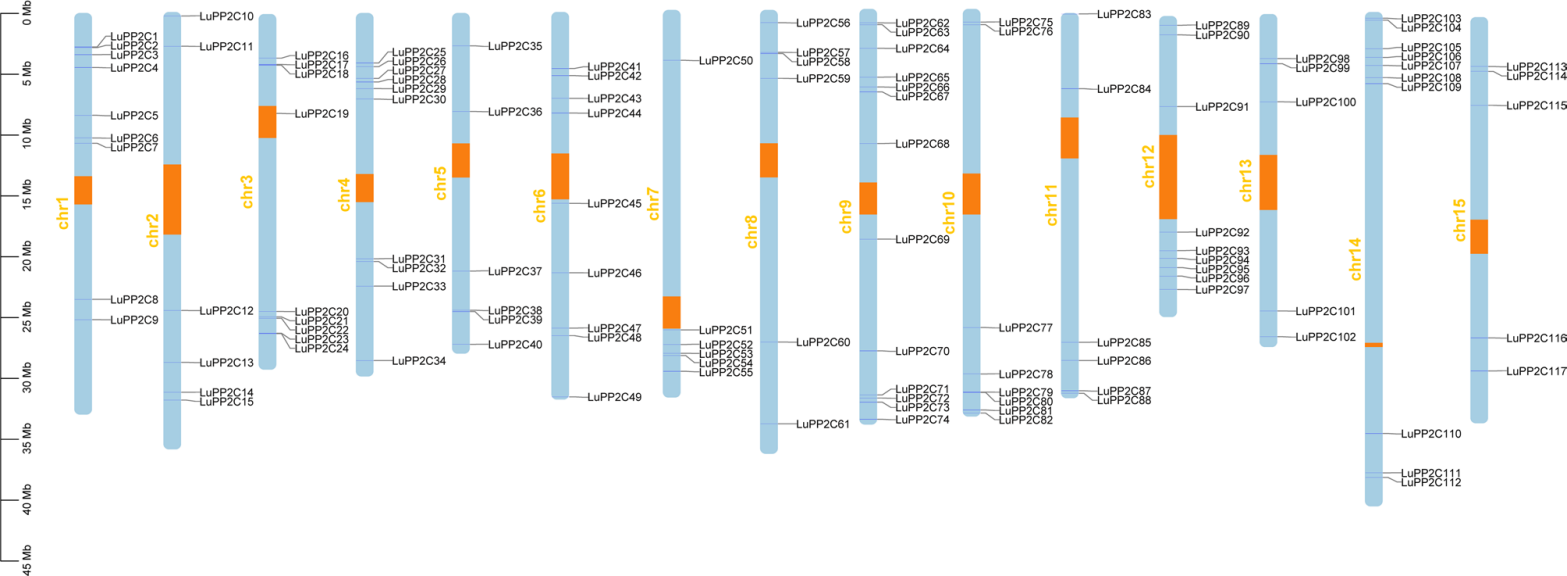
Chromosome distribution map of *LuPP2C* gene. Light blue represents chromosomal regions, while orange represents centromere regions

Chromosomal localization of *LuPP2C* genes was determined based on the flax reference genome, revealing that all 117 *LuPP2C* genes are unevenly distributed across 15 chromosomes (Fig. 3). Chromosome 9 harbored the highest number of *LuPP2C* genes (13 genes, 11.11% of the total), followed by chromosome 4 with 10 genes (8.55%). Chromosomes 13 and 15 contained the fewest *LuPP2C* genes (5 genes each, 4.27% of the total).

**Fig. 4:**
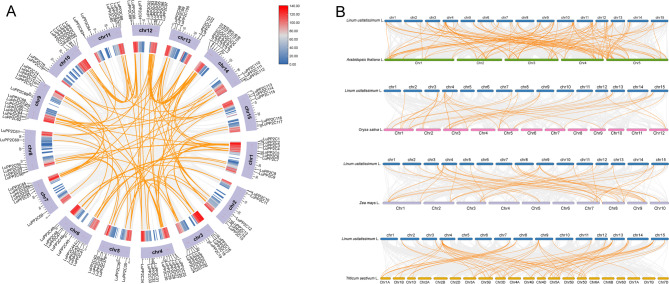
Syntenic relationships of *LuPP2C* genes. **A** Intraspecific synteny of *LuPP2C* genes in flax. **B** Interspecific synteny analysis between flax and *Arabidopsis*, rice, maize and wheat. Orange connectors designate *LuPP2C* orthologous pairs, gray linkages represent genome-wide syntenic blocks

We conducted gene duplication analysis on the *LuPP2C* gene family using BLAST and MCScanX. No tandem repeat sequences were identified within the *LuPP2C* gene family. To explore the gene replication events of the *LuPP2C* gene family, we constructed a Circos map (Fig. 4A). The results revealed the presence of 104 pairs of *LuPP2C* gene pairs in the flax genome, indicating a significant degree of gene family expansion in the flax *PP2C* family. All collinear gene pairs of *LuPP2C* exhibited Ka/Ks ratios below 1, suggesting that they evolved under purifying selection (Table S4). To gain a deeper understanding of the evolutionary relationships between flax and other species, we assessed the homologous relationships between flax and four representative species (*Arabidopsis*, rice, maize, and wheat) (Fig. 4B), aiming to clarify the evolutionary disparities among *PP2C* genes. The findings indicated the existence of 108, 33, 40, and 55 pairs of collinear gene pairs between flax and the four species, respectively. Notably, five chromosomes in *Arabidopsis* exhibit collinearity with all chromosomes in flax. *PP2C* collinear genes located on chromosomes 1, 2, 3, 5, 7, 9, and 12 of rice are found on chromosomes 2–3 and 7–15 of flax. Similarly, maize *PP2C* collinear gene pairs are present on chromosomes 2–3 and 7–15 of flax, while wheat *PP2C* collinear gene pairs are located on chromosomes 2–4, 6–9, and 11–15 of flax. In conclusion, while most *PP2C* genes across flax and the four representative species exhibit conserved collinearity across chromosomal regions, notable differences in gene pairs also exist, underscoring the significant role played by the *LuPP2C* gene family in evolution.

### Analysis of cis-acting elements and prediction of MiRNA

**Fig. 5:**
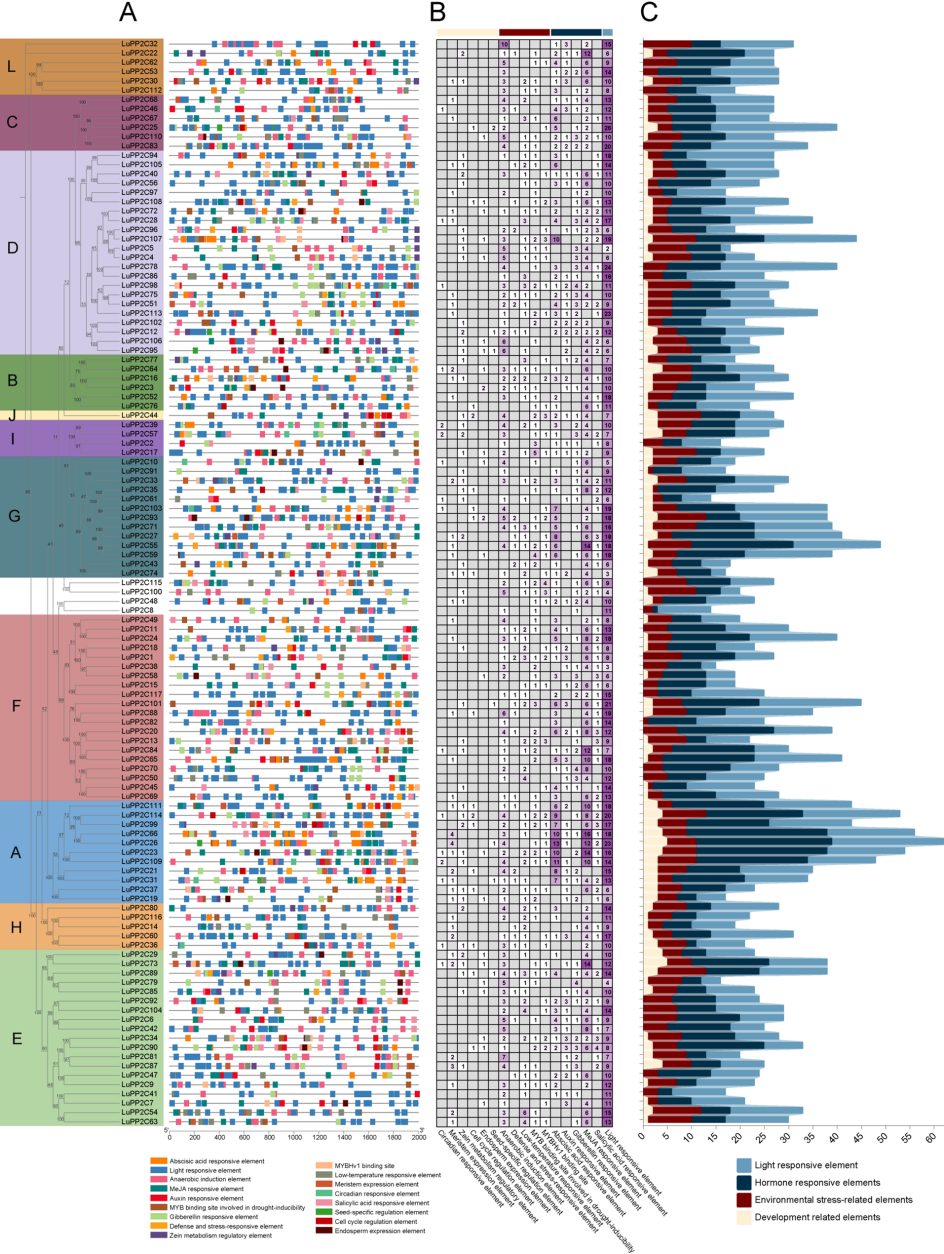
Cis-Acting Element Profiling of *LuPP2C* Genes. **A** Distribution of cis-acting elements across *LuPP2C* subfamilies. **B**, **C** Statistics of cis-acting quantity of *LuPP2C* gene. Light white represents developmental related elements; Deep red represents elements related to environmental stress; Deep blue represents hormone related elements; Light blue represents light responsive elements

To decipher the regulatory mechanisms of the *LuPP2C* gene family in response to abiotic stress, this study analyzed cis-acting elements within the 2 kb promoter regions upstream of these genes (Fig. 5A; Table S5). After excluding core promoter elements such as TATA-box and CAAT-box, a total of 3,328 functional cis-regulatory elements were identified and categorized into four major classes: light-responsive elements (41.20%, 1,371 elements), predominantly represented by G-box, Box 4, GT1-motif, and TCT-motif, suggesting crosstalk between light signaling and stress responses; hormone-responsive elements (34.62%, 1,152 elements), with methyl jasmonate (MeJA)-responsive motifs (TGACG/CGTCA, 687 elements) and abscisic acid (ABA)-responsive ABRE elements (283 elements) being the most abundant, followed by gibberellin-related motifs (P-box/GARE-motif, 162 elements), while auxin- and salicylic acid-associated elements (TGA/AuxRR-core/TCA-element) accounted for only 0.6%, indicating the dominance of MeJA and ABA signaling in *LuPP2C* regulation; stress-responsive elements (18.60%, 619 elements), encompassing hypoxia (ARE/GC-motif), low-temperature (LTR), MYB-mediated drought response (MBS/CCAAT-box), and pathogen defense (TC-rich repeats) pathways; and development-related elements (5.59%, 186 elements), including meristem-specific CAT-box, endosperm expression-associated GCN4_motif, and seed maturation regulator RY-element. Notably, stress- and hormone-responsive elements collectively constituted 93.42% of the total, significantly surpassing developmental elements, implying that evolutionary selection on *LuPP2C* promoters prioritizes enhanced adaptability to environmental fluctuations over fine-tuned developmental regulation. This cis-element profile provides critical insights into the molecular mechanisms by which flax achieves abiotic stress adaptation through the PP2C phosphatase family.

The miRNA prediction results indicated that among the 117 *LuPP2C* genes, only 28 family members (23.93%) were predicted to have 52 miRNA targets (Table 1). The *LuPP2C88* gene exhibited the highest number of miRNA targets (10 targets), including lus-miR166a/c/d/e/f/g/h/j and lus-miR166i/k. Ten genes (*LuPP2C4*,* 5*,* 8*,* 32*,* 39*,* 48*,* 98*,* 104*,* 106*) had the fewest targets, each containing only 1 miRNA target. We observed that a single *LuPP2C* gene could be targeted by multiple miRNAs; for example, *LuPP2C108* was simultaneously targeted by lus-miR395 and lus-miR399. Conversely, distinct *LuPP2C* genes could be targeted by the same miRNA: lus-miR166 targeted three genes (*LuPP2C8*, *LuPP2C48*, *LuPP2C88*), while lus-miR394 targeted four genes (*LuPP2C67*, *LuPP2C79*, *LuPP2C93*, *LuPP2C103*). These findings highlight lus-miR395 as the predominant miRNA targeting the *LuPP2C* gene family.

**Table 1:** Potential MiRNA targets of *LuPP2C* gene

MiRNA	Target	Expectation	MiRNA Length	Target_start	Target_end	Inhibition	Multiplicity
lus-miR159a	*LuPP2C80*	5	20	949	969	Cleavage	1
lus-miR159b/c	*LuPP2C44*	5	20	2243	2262	Cleavage	1
lus-miR160a/b/d/e/f/h/i/j	*LuPP2C63*	5	20	462	481	Translation	1
lus-miR166a/c/d/e/f/g/h/j	*LuPP2C88*	5	20	285	305	Translation	1
lus-miR166b	*LuPP2C8*	5	20	513	532	Translation	1
lus-miR166b	*LuPP2C48*	5	20	513	532	Translation	1
lus-miR166i/k	*LuPP2C88*	5	21	284	305	Translation	1
lus-miR168a/b	*LuPP2C73*	5	20	178	198	Cleavage	1
lus-miR169e/i	*LuPP2C26*	5	20	485	505	Cleavage	1
lus-miR171i	*LuPP2C39*	5	20	966	986	Cleavage	1
lus-miR172e/g/i	*LuPP2C49*	5	20	864	883	Cleavage	1
lus-miR319a	*LuPP2C44*	5	20	2242	2261	Cleavage	1
lus-miR319b	*LuPP2C44*	5	19	2243	2261	Cleavage	1
lus-miR394a/b	*LuPP2C103*	5	19	46	65	Cleavage	1
lus-miR394a/b	*LuPP2C93*	5	19	46	65	Cleavage	1
lus-miR394a/b	*LuPP2C79*	5	19	199	218	Translation	1
lus-miR394a/b	*LuPP2C67*	5	19	1314	1333	Cleavage	1
lus-miR395a/b/c/d	*LuPP2C7*	5	20	957	977	Cleavage	1
lus-miR395a/b/c/d	*LuPP2C22*	5	20	1196	1216	Cleavage	1
lus-miR395e	*LuPP2C104*	5	20	162	182	Cleavage	1
lus-miR395e	*LuPP2C108*	5	20	333	353	Cleavage	1
lus-miR395e	*LuPP2C97*	5	20	330	350	Cleavage	1
lus-miR395e	*LuPP2C4*	5	20	1157	1177	Cleavage	1
lus-miR395e	*LuPP2C5*	5	20	1157	1177	Cleavage	1
lus-miR396a/b/c/e	*LuPP2C9*	5	20	1009	1029	Cleavage	1
lus-miR397b	*LuPP2C106*	5	20	882	902	Cleavage	1
lus-miR397b	*LuPP2C98*	5	20	945	965	Cleavage	1
lus-miR397b	*LuPP2C32*	5	20	2592	2612	Cleavage	1
lus-miR397b	*LuPP2C22*	5	20	2592	2612	Cleavage	1
lus-miR399b/d	*LuPP2C108*	5	20	194	214	Cleavage	1
lus-miR399b/d	*LuPP2C97*	5	20	191	211	Cleavage	1
lus-miR399a/c/e/f/g	*LuPP2C28*	5	20	90	110	Cleavage	1
lus-miR408a	*LuPP2C102*	5	20	369	389	Translation	1
lus-miR408a	*LuPP2C12*	5	20	369	389	Translation	1

### Protein–protein interaction network, GO enrichment analysis, and protein interaction validation of PP2C genes in flax

**Fig. 6:**
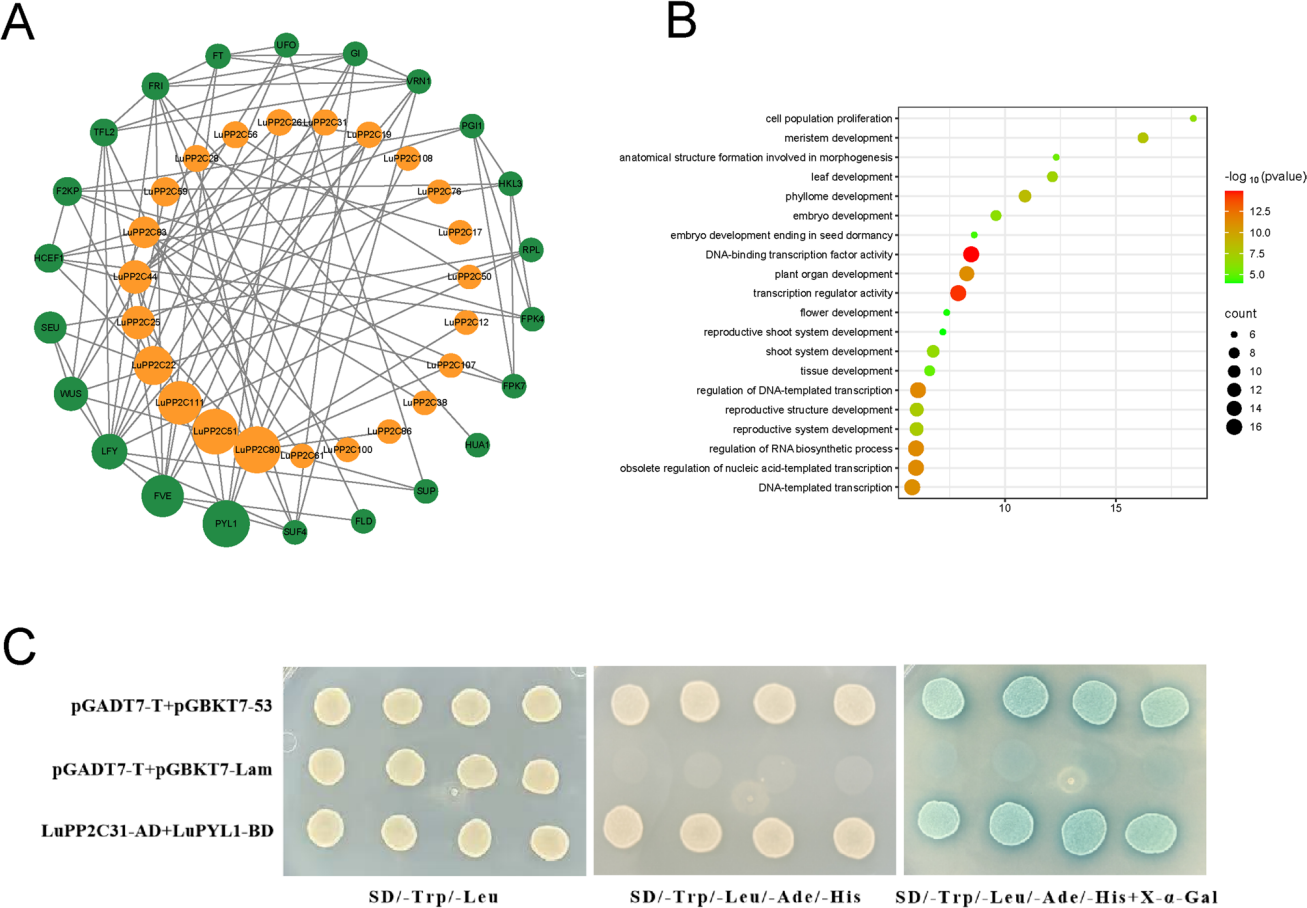
Protein–protein interaction network, GO enrichment analysis, and protein interaction validation of *PP2C* genes in flax. **A** The *LuPP2C* gene family operates through a protein interaction network of homologous genes in *Arabidopsis*. Green represents resistance related genes in *Arabidopsis*, while orange represents the *LuPP2C* gene family (**B**) GO enrichment analysis of the *LuPP2C* gene family. **C **Verification of the interaction between *LuPP2C31* and *LuPYL1*. Experimental group: LuPP2C31-AD + LuPYL1-BD; positive control: pGADT7-T + pGBKT7-53; negative control: pGADT7-T + pGBKT7-LAM

To elucidate the biological functions and regulatory networks of the *LuPP2C* gene family, protein-protein interaction networks were predicted using *Arabidopsis* homologous PP2C proteins as a reference (Fig. 6A). The analysis revealed that only 11 *LuPP2C* genes exhibited interactions with 6 functional *Arabidopsis* genes. Notably, some interacting proteins were associated with stress-responsive mechanisms: PYL1 (a typical ABA receptor involved in drought, salinity, heat, and cold stress responses), FVE (associated with temperature response), and LFY/SEU (related to meristem differentiation and floral development). Gene Ontology enrichment analysis of *LuPP2C* genes in flax highlighted their predominant roles in biological processes such as cell population proliferation, meristem development, DNA-binding transcription factor activity, plant organ development, and transcription regulator activity.

Based on protein interaction network analysis, the two proteins with the highest correlation were selected for yeast two-hybrid validation. We first identified the homologous gene of *AtPYL1*, LuPYL1, and verified the interaction between *LuPP2C31* and *LuPYL1* proteins. The *LuPP2C31* gene was inserted into the pGADT7 vector, and the *LuPYL1* gene was inserted into the pGBKT7 vector. The recombinant vectors were transformed into yeast competent cells and cultured at 30 °C on SD (− Trp/−Leu), SD (− Trp/−Leu/−Ade/−His), and SD (− Trp/−Leu/−Ade/−His) + X-α-gal media. The Y2H results showed that yeast cells co-transformed with LuPP2C31-AD and LuPYL1-BD grew normally on SD (− Trp/−Leu/−Ade/−His) medium, indicating an interaction between LuPP2C31 and LuPYL1 proteins in yeast cells.

### Expression pattern analysis of LuPP2C gene family

**Fig. 7:**
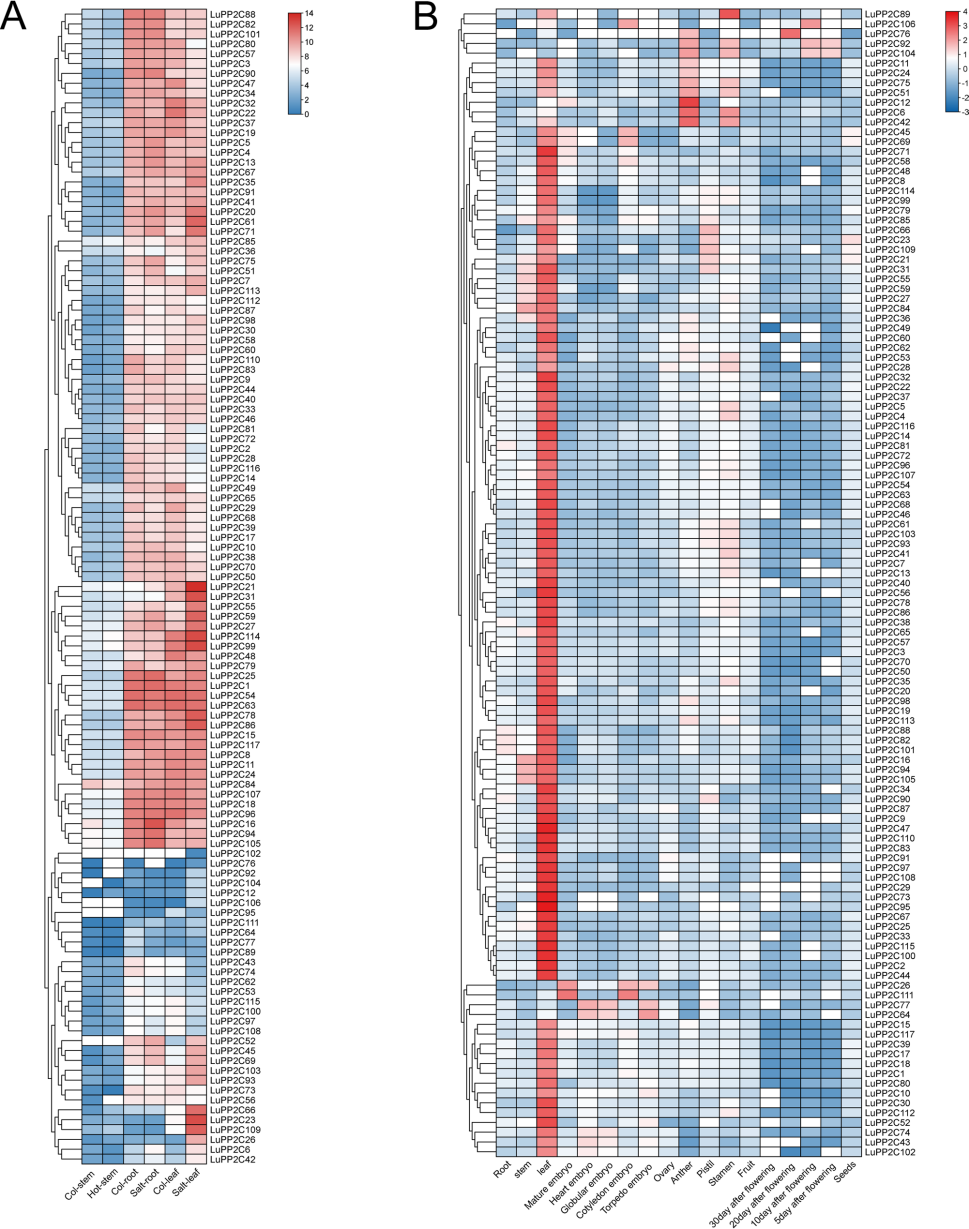
Expression pattern analysis of *LuPP2C* gene family. **A** The expression patterns of *LuPP2C* gene under salt and heat treatment. **B** Tissue-specific expression atlas in flax. Expression levels are log2(FPKM + 1) transformed, visualized via gradient color scaling from high (red) to low (blue)

To explore the potential functions of *LuPP2C* genes in responding to abiotic stress, we analyzed their expression patterns under salt and heat stress using publicly available transcriptomic data (Fig. 7A). The *LuPP2C* gene family exhibited no significant changes under heat stress, with only *LuPP2C16* and *LuPP2C48* showing downregulated expression. In contrast, most *LuPP2C* genes displayed marked expression changes in salt-stressed leaf and root tissues. Compared to the control, 11 genes (*LuPP2C16*,* 20*,* 21*,* 27*,* 45*,* 51*,* 52*,* 59*,* 69*,* 71*,* 75*) were upregulated in salt-stressed roots, with *LuPP2C16* showing the most pronounced differential expression. In salt-stressed leaves, 30 genes (*LuPP2C6*,* 13*,* 20–23*,* 26*,* 27*,* 31*,* 35*,* 41*,* 45*,* 51*,* 52*,* 55*,* 59*,* 61*,* 66*,* 67*,* 69*,* 71*,* 75*,* 78*,* 86*,* 98*,* 99*,* 103*,* 109*,* 113*,* 114*) were significantly upregulated, with *LuPP2C21* and *LuPP2C23* exhibiting the highest expression differences.

To gain deeper insights into the molecular mechanisms underlying the *LuPP2C* gene family’s regulatory roles in plant growth and development, we systematically analyzed the spatiotemporal expression profiles of all identified *LuPP2C* genes across 18 functionally distinct tissues in flax using RNA-seq data. (Fig. 7B). The expression profiles of *LuPP2C* genes varied significantly among tissues. Results showed that most *LuPP2C* genes were highly expressed in leaf tissues, while exhibiting low expression in roots, ovaries, pistils, fruits, flower tissues at 30 days post-anthesis (DPA), and seeds. Four members (*LuPP2C16*,* LuPP2C84*,* LuPP2C94*,* LuPP2C105*) showed high expression in stem tissues. Only two genes (*LuPP2C26*,* LuPP2C111*) were highly expressed in mature embryos, whereas two members (*LuPP2C64*,* LuPP2C77*) exhibited elevated expression in both heart-shaped embryos and globular embryos. Five members (*LuPP2C26*,* LuPP2C45*,* LuPP2C69*,* LuPP2C106*,* LuPP2C111*) were highly expressed in cotyledon-stage embryos, and three members (*LuPP2C26*,* LuPP2C64*,* LuPP2C77*) showed high expression in torpedo-stage embryos. In anthers, seven members (*LuPP2C6*,* LuPP2C12*,* LuPP2C42*,* LuPP2C51*,* LuPP2C75*,* LuPP2C76*,* LuPP2C104*) were upregulated. Three genes (*LuPP2C6*,* LuPP2C42*,* LuPP2C89*) were highly expressed in stamens, while *LuPP2C76* was the sole gene upregulated in flower tissues at 20 DPA. Both LuPP2C92 and LuPP2C104 showed high expression in flower tissues at 10 DPA and 5 DPA. In summary, the predominant high expression of *LuPP2C* genes in leaf tissues suggests that flax leaves may serve as the primary site for regulating reactive oxygen species homeostasis and act as the first line of antioxidant defense in plants, with *LuPP2C* genes playing critical regulatory roles in these processes.

### Expression level of LuPP2C gene in flax under abiotic stress

**Fig. 8:**
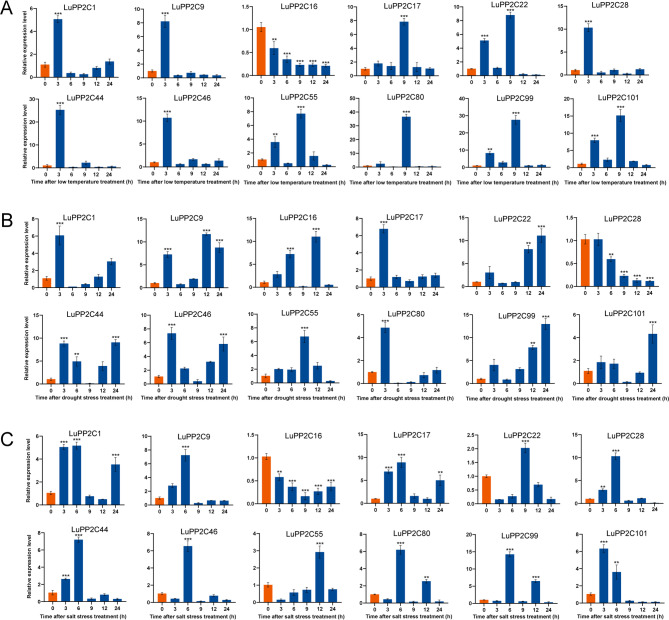
Abiotic Stress-Responsive Expression Profiling of *LuPP2C* Genes. **A** Transcriptional dynamics under low-temperature stress. **B** Drought-induced expression modulation. **C** Salinity stress response. Histograms depict normalized expression levels log^2^(FPKM + 1), with untreated controls (orange) and stress-treated samples (blue). Statistical significance (Student’s t-test) is denoted by asterisks: **p < 0.01; ***p < 0.001

To ensure that the selected genes represent the diversity of the *LuPP2C* family, we selected 12 genes from 11 subfamilies that have high homology with known genes in *Arabidopsis*. In addition, qRT PCR was used to evaluate the relative expression levels of these selected genes to determine whether the *LuPP2C* gene responds to abiotic stress. And the expression levels of *LuPP2C* gene in leaves at 0 h, 3 h, 6 h, 9 h, 12 h, and 24 h were detected, and the relative expression levels of the 0 h control group were compared (Fig. 8). With the increase of low temperature stress time, all *LuPP2C* genes except *LuPP2C16* showed significant responses (Fig. 8A). Among them, five genes (*LuPP2C1*,* LuPP2C9*,* LuPP2C28*,* LuPP2C44*, and *LuPP2C46*) reached their peak expression after 3 h of low temperature stress, and then rapidly decreased. Compared to the control group, they increased by approximately 4.1 times, 7.2 times, 9.3 times, 25.3 times, and 10.7 times, respectively. The expression of *LuPP2C17* and *LuPP2C80* genes reached their peak at 9 h of low temperature stress, with an increase of approximately 7.9 times and 36.6 times, respectively, compared to the control group. *LuPP2C22*, *LuPP2C55*, *LuPP2C99*, and *LuPP2C101* were significantly upregulated at 3 and 9 h of low temperature stress. With the increase of drought stress time, the expression level of *LuPP2C* gene showed significant changes (Fig. 8B). Six genes (*LuPP2C1*,* LuPP2C9*,* LuPP2C17*,* LuPP2C44*,* LuPP2C46*, and *LuPP2C80*) were significantly upregulated under drought stress, with increases of approximately 6.1 times, 7.3 times, 6.8 times, 8.8 times, 7.4 times, and 4.9 times, respectively, compared to the control group. Three genes (*LuPP2C9*, *LuPP2C22*, and *LuPP2C99*) were significantly upregulated at 12 and 24 h of drought stress, with increases of approximately 11.7 and 8.4 times, 8.1 and 11.1 times, 7.9 and 13 times, respectively, compared to the control group. With the increase of salt stress time, the *LuPP2C* gene showed a significant response (Fig. 8C). Among them, 8 genes (*LuPP2C1*,* LuPP2C9 LuPP2C17*,* LuPP2C28*,* LuPP2C44*,* LuPP2C46*,* LuPP2C80*,* LuPP2C9*9) were significantly upregulated and reached their peak in leaf tissues under salt stress for 6 h, with increases of about 5.2 times, 7.2 times, 8.9 times, 10.3 times, 7.2 times, 6.5 times, 6.2 times, and 14.2 times compared to the control group, respectively. *LuPP2C22* and *LuPP2C55* were significantly upregulated under salt stress for 9 h and 12 h, respectively, with an increase of approximately 2-fold and 2.9-fold compared to the control group. In summary, the *LuPP2C* gene showed a significant response at 6 h under salt stress, which may also be a key time point for the *PP2C* gene to respond to salt stress.

### Expression levels of LuPP2C A subfamily genes under abiotic stress

**Fig. 9:**
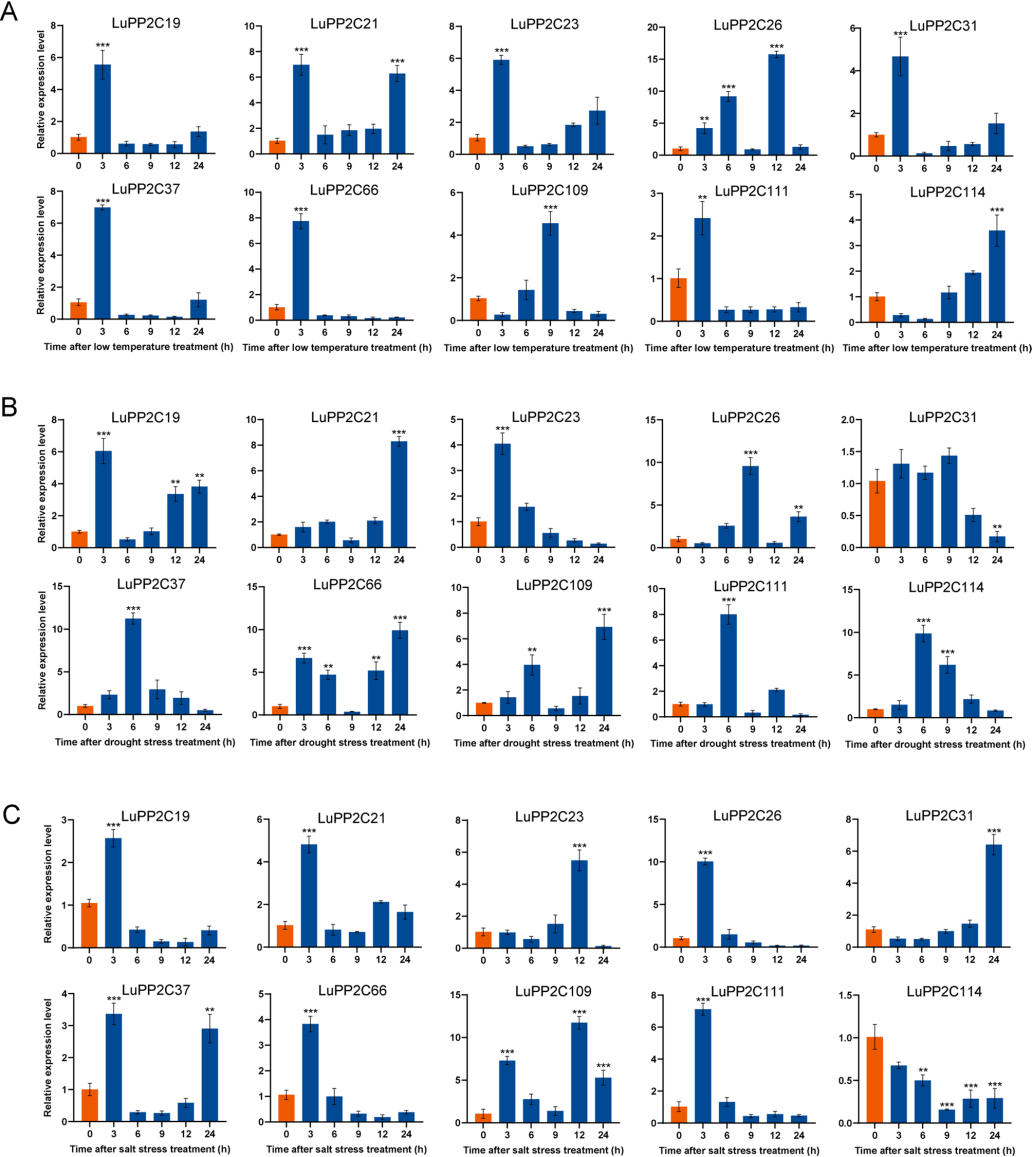
Analysis of expression patterns of subfamily A in the *LuPP2C* gene family under abiotic stress. **A** Analysis of expression patterns under cold stress (**B**) Expression patterns under drought stress. **C** Expression patterns under salt stress. The orange column represents the control group, and the blue column represents the treatment group. Using Student’s t-test, asterisks indicate statistically significant differences between the treatment group and the control group. (**p < 0.01; ***p < 0.001)

In previous experiments, it was found that the A subfamily of the *LuPP2C* gene family has a positive response to abiotic stress (cold, drought, and salt). The relative expression levels of 10 *LuPP2C* genes in the A subfamily were evaluated in leaf tissues using qRT-PCR (Fig. 9). With the increase of low temperature stress time, all *LuPP2C* genes showed significant responses (Fig. 9A). Seven genes (*LuPP2C19*,* LuPP2C21*,* LuPP2C23*,* LuPP2C31*,* LuPP2C37*,* LuPP2C66*, and *LuPP2C111*) reached their maximum values under low temperature stress for 3 h, increasing by 5.5 times, 7 times, 5.9 times, 4.7 times, 7 times, 7.7 times, and 2.4 times respectively compared to the control. As the duration of drought stress increased, all *LuPP2C* genes showed significant responses (Fig. 9B). *LuPP2C37*, *LuPP2C111*, and *LuPP2C114* reached their maximum values under drought stress for 6 h, increasing by 11.3 times, 8 times, and 9.9 times respectively compared to the control. *LuPP2C21*, *LuPP2C66*, and *LuPP2C109* reached their maximum values under drought stress for 24 h, increasing by 8.3 times, 9.9 times, and 6.9 times respectively compared to the control. With the increase of salt stress time, most LuPP2C showed significant response at 3 h (Fig. 9B). Among them, six genes (*LuPP2C19*, *LuPP2C21*, *LuPP2C26*, *LuPP2C37*, *LuPP2C66*, and *LuPP2C111*) reached their extremum at 3 h of salt stress, increasing by 2.6 times, 4.8 times, 10.1 times, 3.4 times, 3.8 times, and 7.1 times respectively compared to the control.

### Salt tolerance test of yeast transformed bacteria

**Fig. 10:**
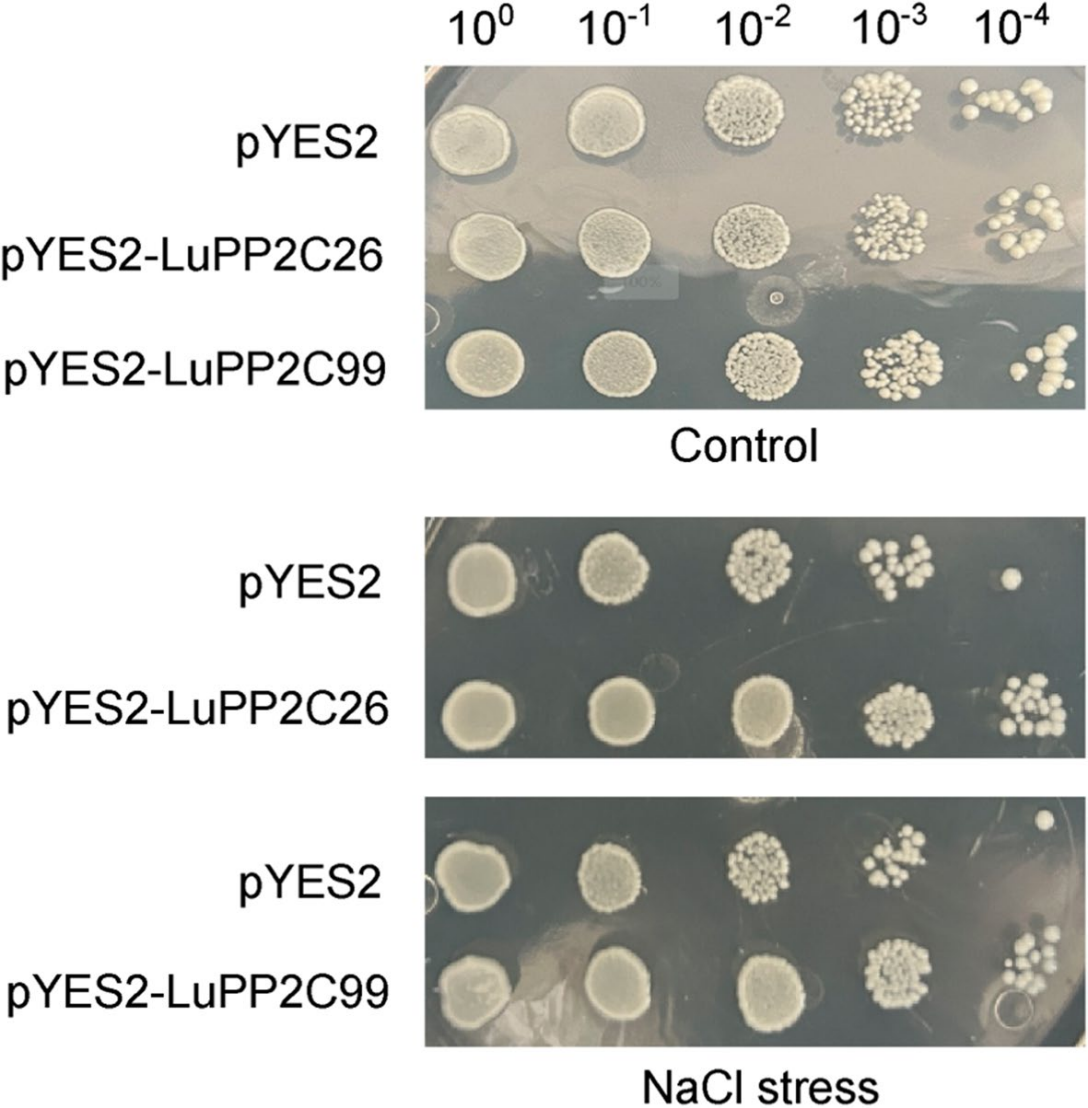
Overexpression of *LuPP2C26* and *LuPP2C99* enhanced salt stress tolerance in transformed yeast. Control group yeast was grown at 30 °C for 48 h under normal conditions, while treatment group yeast were cultured under the same temperature and duration but with 1 M NaCl to simulate salt stress

*LuPP2C26* and *LuPP2C99* were selected as candidate genes for further study based on previous experiments. To determine the effect of their proteins on the survival rate of yeast recombinants under salt stress, the growth of yeast colonies carrying the pYES2-*LuPP2C26* and pYES2-*LuPP2C99* vectors, as well as the control strain containing the empty vector (pYES2), was examined under 1 M NaCl stress (Fig. 10). The results showed that under optimal conditions, there was no significant difference in growth between the transformed yeast and the control yeast. However, under 1 M NaCl salt stress, the transformed yeast exhibited stronger growth compared to the control group. This indicates that overexpression of *LuPP2C26* and *LuPP2C99* enhances the salt tolerance of yeast transformants.

## Discussion

*PP2C* plays a crucial role in fundamental functions such as plant hormone signaling, development, and responses to abiotic stress [39, 40]. Although *PP2C* has been reported in other species, information on PP2C in flax remains limited. To date, no studies have identified the *LuPP2C* gene family in flax using a genomic approach. In this study, 117 *LuPP2C* genes were identified in the flax T2T genome (Table S1). The flax genome contains a significantly higher number of LuPP2C genes (117) compared to cucumber (56) [21] and rubber tree (60) [41]. It is marginally higher than the PP2C counts in maize (102) [42] and *Cucurbita pepo* (102) [43], while being lower than those in soybean (134) [44] and peanut (178) [45]. The phylogenetic analysis classified the *LuPP2C* genes into 12 subfamilies (Fig. 1), which is largely consistent with previous studies [15]. Flax was an ancient diploid plant that underwent three whole-genome duplication (WGD) events approximately 11.5, 53.5, and 114 million years ago (MYA) [46]. WGD was likely one of the primary driving forces behind the expansion of the LuPP2C gene family, providing a large number of redundant gene copies.

According to the conserved motif analysis, most *LuPP2C* proteins exhibit a similar motif arrangement (Fig. 2B). This pattern consists of the sequence motif3, motif2, motif6, motif5, motif1, and motif9, with only motifs 1–4 being associated with the PPM-type phosphatase domain. This finding is consistent with studies in jute [15]. Gene structure analysis revealed that the number of exons in *LuPP2C* genes ranges from 1 to 22, while the number of introns varies from 0 to 21 (Fig. 2C). However, most genes exhibit structural differences. These variations in *PP2C* gene structure may be attributed to exon/intron insertion or deletion mechanisms [47]. We found that each *LuPP2C* subfamily exhibits a conserved exon-intron motif pattern, suggesting that *LuPP2C* genes play similar roles in various abiotic stress-related responses. This finding is consistent with observations in *Brassica napus* [48].

Gene duplication events played a pivotal role in the evolutionary diversification of PP2C genes, as duplicated paralogs frequently underwent neofunctionalization, thereby enhancing plant adaptive capacity to environmental stressors [49]. In this study, 117 *LuPP2C* genes were unevenly distributed across 15 chromosomes, with no tandem duplications detected (Fig. 3). However, 104 pairs of segmentally duplicated collinear genes were identified (Fig. 4A), indicating that segmental duplication is the primary driver of *LuPP2C* gene expansion [50]. Collinearity analysis is a powerful method for analyzing the evolutionary trajectory of genes [50]. In this study, 108, 33, 40, and 55 pairs of co-linear gene pairs between the flax *PP2C* gene and *Arabidopsis*, rice, maize, and wheat, respectively, are shared (Fig. 4B). It is suggested that these homologous gene pairs may have a common ancestor that existed before divergence [51]. Phylogenetic analysis revealed high evolutionary conservation of the *LuPP2C* genes in flax, with gene family expansion primarily driven by segmental duplication events.

Promoter cis-elements participate in and regulate gene expression. The interaction between transcription factors and promoter binding sites plays a key role [52]. Analysis of the *LuPP2C* promoter regions revealed a predominance of cis-regulatory elements associated with phytohormone signaling and abiotic stress adaptation (Fig. 5). Specifically, methyl jasmonate (MeJA) and abscisic acid (ABA) signaling pathways were prominently represented through TGACG/CGTCA motifs (687 elements) and ABRE motifs (283 elements), respectively, suggesting their central roles in defense activation and drought tolerance. Among abiotic stress-responsive elements, the low-temperature response (LTR) motif mediated cold-inducible transcriptional regulation, while anaerobic response elements (ARE/GC-motifs) at facilitated hypoxia adaptation. Notably, TC-rich repeats positioned distally exhibited strong association with drought resistance, potentially amplifying dehydration signals through chromatin conformational changes. This cis-regulatory landscape highlights evolutionary selection for stress-hormone crosstalk, with 93.42% of elements linked to environmental sensing and adaptive plasticity, reflecting flax’s prioritization of stress resilience over developmental fine-tuning [53]. The promoter of *LuPP2C* contains ABA and MeJA response elements, including ABRE, TGACG-motif, and CGTCA-motif, as well as MYB binding sites (MBS) involved in drought induction. This suggests that these cis-elements are highly conserved and may play a crucial role in regulating stress-related hormones or biotic/abiotic responses. Studies have shown that the promoters of the A subfamily members of *Arabidopsis*, millet, rice, and two-flowered short-beard grass contain more ABRE elements than other subfamily members, and there is evidence that the number of ABRE elements is correlated with gene expression levels after stress [54, 55].

MiRNAs are key regulatory factors that coordinate plant development and plant-environment interactions [56]. Analysis indicated that lus-miR395 was the primary miRNA targeting the *LuPP2C* gene family (Table 2). Studies have shown that in *Arabidopsis*, miR395 became a core component of the plant sulfate assimilation regulatory network through a multi-target synergistic mechanism [57]. In rice, miR395 significantly enhanced the rice’s resistance to two destructive bacterial pathogens by regulating the accumulation and distribution of sulfate [58]. The regulatory mechanisms of the *PP2C* gene and the miRNA159-*PP2C* module need further investigation. Interactions between proteins are crucial for maintaining normal protein function and are key to predicting the functional diversity of proteins [59]. In this study, only 11 *LuPP2C* genes interacted with 6 functional genes in *Arabidopsis* (Fig. 6). These interactions include those with SPY1, FVE, and SEU. Pyrabactin resistance 1-like (PYL) was a family of abscisic acid (ABA) receptor proteins in plants. By inhibiting *PP2C* activity and releasing *SnRK2* kinases, it regulated the expression of downstream transcription factor families such as ABF and WRKY, serving as a central regulatory hub in plant responses to drought stress [60]. In alfalfa, both *MsPYL6* and *MsPYL9* interacted with at least one *MsPP2CA*, thereby improving drought tolerance by regulating stomata [61]. In rapeseed, *BnaPP2C37* interacted with the *BnaPYR/PYL* ABA receptor and negatively regulated drought tolerance [1]. Consistent with previous research findings, in this study, we also confirmed the interaction between *LuPP2C31* and *LuPYL1*. FVE was involved in the regulation of clock genes, affecting plant adaptation to low temperatures, and regulated the CBF signaling pathway to enhance cold tolerance [62]. The SEU gene influenced root hair development by regulating the ethylene synthesis gene ACS4 [63]. *PP2C* genes play an important role in plant growth and development networks, as well as in stress response networks. Therefore, exploring *PP2C* genes and analyzing their interactions with stress responses is crucial for optimizing crop production performance.

Analysis of RNA-seq data revealed tissue-specific expression patterns among *LuPP2C* gene family members, with most genes exhibiting high transcriptional activity in leaves (Fig. 7B). The *ZmPP2C* gene family in maize showed diverse tissue-specific expression patterns, indicative of possible subfunctionalization or neofunctionalization among paralogous gene pairs. For instance, *ZmPP2C42* and *ZmPP2C47* were predominantly expressed in mature pollen, whereas *ZmPP2C59* was specifically expressed in primary roots and root cortex tissues [42]. In the cucumber *CsPP2C* gene family, with the exception of a few members such as *CsPP2C11*, *CsPP2C41*, *CsPP2C5*, *CsPP2C33*, and *CsPP2C50*, the majority of members exhibited significantly high expression levels in fertilized ovules, male flowers, female flowers, and leaf tissues, while their expression levels in other organs or tissues were relatively low [21]. These results suggest that *PP2C* genes play an important regulatory role in plant growth and development, with some *PP2C* genes having specific functions in particular tissues. These findings across species suggest that *PP2C* genes may exert tissue-specific functions during plant development. In flax, the predominant expression of *LuPP2C* genes in leaves may indicate a key regulatory role in maintaining redox balance and mitigating abiotic stress, such as salinity and drought, especially given the leaves’ exposure to external environmental factors. Leaves are not only the primary site of photosynthesis but also serve as a major platform for stress perception and signaling. In contrast, although *LuPP2C* genes generally exhibited low expression in roots under non-stress conditions, several members were strongly induced under salt stress, suggesting that their functions in roots may be stress-inducible rather than constitutive. Overall, the tissue-specific analysis of flax provides deeper insights into the functional study of the *LuPP2C* gene.

The PP2C phosphatase family, ubiquitously conserved across plant species, serves as a multifunctional regulatory module. In flax, *LuPP2C* genes not only modulate developmental programming but are critically involved in stress signaling cascades. Transcriptional profiling under salt stress revealed significant upregulation of 11 *LuPP2C* paralogs in root tissues compared to untreated controls, with *LuPP2C16* demonstrating the most pronounced differential expression profile (Fig. 7A). In flax leaf tissues, 30 genes were significantly upregulated under salt stress. Through qRT-PCR validation, we observed that under cold stress, five genes (*LuPP2C1*, *LuPP2C9*, *LuPP2C28*, *LuPP2C44*, and *LuPP2C46*) reached peak expression levels at 3 h of low-temperature exposure, followed by rapid decline. Under drought stress, six genes (*LuPP2C1*, *LuPP2C9*, *LuPP2C17*, *LuPP2C44*, *LuPP2C46*, and *LuPP2C80*) were significantly upregulated. Notably, under salt stress, eight genes (*LuPP2C1*, *LuPP2C9*, *LuPP2C17*, *LuPP2C28*, *LuPP2C44*, *LuPP2C46*, *LuPP2C80*, and *LuPP2C99*) displayed significant upregulation and achieved peak expression levels (Fig. 8). These findings suggest that *LuPP2C* genes may function as key regulators in plant stress adaptation. Previous studies found that the expression levels of the peanut *AhPP2C45* and *AhPP2C134* genes were significantly upregulated under salt stress [45]. Microarray analysis of *OsNAP*-overexpressing transgenic rice plants subjected to high salt, drought, and low-temperature treatments revealed that the expression of the stress-associated gene *OsPP2C06* was significantly upregulated [64]. *BnPP2C1*, *BnPP2C26*, and *BnPP2C27* in ramie were activated by drought, salinity, and ABA [65]. These findings provide strong evidence that *PP2C* genes play crucial roles in abiotic stress responses in flax. Furthermore, qRT-PCR analysis of the *PP2C* subfamily A demonstrated that seven genes (*LuPP2C19*, *LuPP2C21*, *LuPP2C23*, *LuPP2C31*, *LuPP2C37*, *LuPP2C66*, and *LuPP2C111*) reached peak expression levels at 3 h of cold stress, while *LuPP2C37*, *LuPP2C111*, and *LuPP2C114* peaked at 6 h of drought stress. Additionally, six genes (*LuPP2C19*, *LuPP2C21*, *LuPP2C26*, *LuPP2C37*, *LuPP2C66*, and *LuPP2C111*) exhibited maximum expression at 3 h under salt stress (Fig. 9). Salt tolerance assays in yeast transformants demonstrated that overexpression of *LuPP2C26* and *LuPP2C99* significantly enhanced salt tolerance in the transgenic yeast strains (Fig. 10). The A subfamily genes of two-flowered short-beard grass exhibited significant responses to abiotic stress. Exogenous ABA treatment significantly induced the expression of 6 genes in this subfamily, with *BdPP2C* family members (including *BdPP2C36*, *BdPP2C37*, and *BdPP2C44*) showing differential expression patterns under drought stress. Notably, these three genes displayed sustained high-level upregulation across various stress treatments (including osmotic, salt stress, and low-temperature treatments), exhibiting broad stress response characteristics [55]. In rice, all members of the OsPP2C-A subfamily exhibited significant transcriptional upregulation following ABA and salt treatments [54]. These findings strongly support the critical role of *PP2C* genes in abiotic stress adaptation, as evidenced by their conserved functions in flax. Characterization of the *LuPP2C* family lays the groundwork for elucidating the molecular mechanisms underlying *PP2C*-mediated stress resistance in flax.

## Conclusions

This study provides the first genome-wide characterization of the *PP2C* gene family in flax, uncovering 117 members based on a T2T gapless genome. Our integrative analyses revealed that *LuPP2C* genes are phylogenetically diverse and exhibit subfamily-specific regulatory patterns, particularly in response to hormonal cues and abiotic stress signals. Notably, *LuPP2C26* and *LuPP2C99* were experimentally validated to confer salt tolerance in yeast, suggesting their potential functional importance in stress resilience. These findings lay a molecular foundation for dissecting stress signaling pathways in flax and offer promising targets for genetic improvement toward enhanced environmental adaptability.

## Supplementary Information

Supplementary Material 1.

Supplementary Material 2.

Supplementary Material 3.

Supplementary Material 4.

Supplementary Material 5.

Supplementary Material 6.

## Data Availability

Data availability all data generated or analyzed during this study are included in this published article and its supplementary files. The datasets analysed during the current study are available in the NCBI Sequence Read Archive database (project number: PRJNA1002756, PRJNA833557, PRJNA663265, PRJNA977728, and PRJNA874329). Further inquiries can be directed to the corresponding author (jian.zhang@ubc.ca).
